# Transplantation of R-GSIK scaffold with mesenchymal stem cells improves neuroinflammation in a traumatic brain injury model

**DOI:** 10.1007/s00441-020-03247-0

**Published:** 2020-07-27

**Authors:** Sajad Sahab Negah, Mohammad Moein Shirzad, Ghazale Biglari, Farzin Naseri, Hassan Hosseini Ravandi, Ali Hassani Dooghabadi, Ali Gorji

**Affiliations:** 1 Neuroscience Research Center, Mashhad University of Medical Sciences, Mashhad, Iran; 2 Department of Neuroscience, Faculty of Medicine, Mashhad University of Medical Sciences, Mashhad, Iran; 3 Shefa Neuroscience Research Center, Khatam Alanbia Hospital, Tehran, Iran; 4 Student Research Committee, Mashhad University of Medical Sciences, Mashhad, Iran; 5 Department of Neurosurgery and Department of Neurology, Westfälische Wilhelms-Universität, Münster, Germany; 6 Epilepsy Research Center, Westfälische Wilhelms-Universität Münster, Münster, Germany

**Keywords:** Head injury, Stem cells, Nano-scaffold, Neuroinflammation

## Abstract

Neural tissue engineering has been introduced as a novel therapeutic strategy for traumatic brain injury (TBI). Transplantation of mesenchymal stem cells (MSCs) has been demonstrated to improve functional outcome of brain injury, and RADA4GGSIKVAV (R-GSIK), a self-assembling nano-peptide scaffold, has been suggested to promote the behavior of stem cells. This study was designed to determine the ability of the R-GSIK scaffold in supporting the effects of MSCs on motor function activity and inflammatory responses in an experimental TBI model. A significant recovery of motor function was observed in rats that received MSCs+R-GSIK compared with the control groups. Further analysis showed a reduction in the number of reactive astrocytes and microglial cells in the MSCs and MSCs+R-GSIK groups compared with the control groups. Furthermore, western blot analysis indicated a significant reduction in pro-inflammatory cytokines, such as TLR4, TNF, and IL6, in the MSCs and MSCs+R-GSIK groups compared with the TBI, vehicle, and R-GSIK groups. Overall, this study strengthens the idea that the co-transplantation of MSCs with R-GSIK can increase functional outcomes by preparing a beneficial environment. This improvement may be explained by the immunomodulatory effects of MSCs and the self-assembling nano-scaffold peptide.

## Introduction

Traumatic brain injury (TBI) results in a set of secondary pathological alterations within the brain and causes high rates of mortality and disability worldwide (Chauhan 2014; Menon et al. 2010). The secondary injury after TBI includes inflammatory response, which is a key factor in the progression of injury (Helmy et al. 2011; Correale and Villa 2004). Activation of the inflammatory pathways is mediated by the release of pro- and anti-inflammatory cytokines. The inflammatory response in TBI is characterized by the recruitment of peripheral leukocytes into the central nervous system (CNS) and the activation of resident immune cells (Ziebell and Morganti-Kossmann 2010; Rhodes 2011). Activated microglia secrete some cytotoxic factors such as cytokines, reactive oxygen species, nitric oxide, and proteases, which may initiate neuronal death (Lucas et al. 2006; d’Avila et al. 2012).

Despite many attempts in the last decades, no definite effective treatment has been identified for TBI (Skolnick et al. 2014; Wright et al. 2014). During the last decades, tissue engineering has emerged as a novel strategy to restore the injured tissue in TBI (Zhou et al. 2018). The essential part of this method is constructing new tissue substitutes composed of biological scaffolds and stem cells to improve the recovery and preservation of injured tissue (Zhou et al. 2018). Among different types of scaffolds, self-assembling peptides (SAPs) not only present a three-dimensional environment for cell adhesion, growth, and migration but also create suitable micro-environments for the nutrition and waste excretion of cells (Sahab Negah et al. 2019). RADA16 (Ac-RADA RADA RADA RADA-CONH2) is one of the SAPs, which has been demonstrated to improve stem cell behavior in both in vitro and in vivo experiments (Sahab Negah et al. 2018). RADA16 significantly improved stem cell survival, migration, and differentiation when conjugated with functional motifs (Sahab Negah et al. 2019; Shi et al. 2016). IKVAV (isoleucine-lysine-valine-alanine-valine), a functional motif of the laminin molecule, improves stem cell survival after transplantation in TBI models (Sahab Negah et al. 2018). Therefore, we developed a biodegradable scaffold containing RADA and IKVAV (RADA16GGSIKVAV; R-GSIK) (Sahab Negah et al. 2017). R-GSIK has shown great potential in the improvement of adhesion, proliferation, viability, and differentiation of cells, and stem cells show a greater survival rate when implanted R-GSIK scaffold in rats subjected to TBI (Sahab Negah et al. 2017, 2019).

On the other hand, mesenchymal stem cells (MSCs) have a good potential in functional differentiation into neuron-like cells and act as immunomodulatory and regenerative substances (Munoz et al. 2012; Zeng et al. 2013; Cho et al. 2005). Furthermore, MSCs are a good candidate for clinical applications because of their readily accessible tissue sourced for autologous transplantation (Galipeau and Sensébé 2018). MSCs modulate the secondary mechanisms of injury and cease the development of the secondary pathways in TBI (Zhang et al. 2013). MSCs also secrete growth factors that facilitate the regeneration of neural tissue (Mahmood et al. 2004). Recently, several studies have shown that MSCs improved motor function in different animal models of TBI (Yang et al. 2017; Hasan et al. 2017). However, only a few studies have focused on the effects of MSCs with nano-scaffold on immune cells and inflammation-associated cytokines in brain injury (Zhou et al. 2018; Shi et al. 2016). This study aimed to investigate the effect of the combination of MSCs and R-GSIK on functional recovery as well as neuroinflammatory responses after TBI.

## Materials and methods

### Study design

In this study, MSCs were isolated from rat abdominal adipose tissue and cultured in an adherent plastic flask. MSCs were characterized and transplanted in a rat TBI model with/without a three-dimensional nano-scaffold. To detect sensory-motor activity and anxiety-like behavior, we performed a modified neurological severity score (mNSS), open field (OF), and elevated plus maze (EPM) at different predetermined time points. To determine the efficacy of co-transplantation of MSCs and nano-scaffold, pro-inflammatory cytokines and reactive glia were investigated 30 days after TBI.

### Ethical statement

All experimental procedures were approved by the Mashhad University of Medical Sciences Animal Experimentation Ethical Committee. Rats were handled before behavioral testing to eliminate the anxiety resulting from human contact. To prevent potential biases of performance and detection, all experiments were performed by a person who was blinded to the experimental groups.

### Isolation and culture of MSCs

Adipose tissue from adult male rats was removed under sterile conditions. Adipose tissues were washed and suspended in phosphate buffer saline (PBS) containing 5% penicillin/streptomycin (P/S). Then, the samples were minced by a scalpel and digested using 0.075% collagenase type I and incubated for 30 min at 37 °C, 5% CO2. Fetal bovine serum (FBS) was used to neutralize the Collagenase Type I. The samples were then centrifuged two times at 2000 rpm for 5 min. Then, the samples were washed with PBS and then centrifuged for 5 min at 2000 rpm. The MSCs were cultured with a stromal medium containing 20% FBS, 1% l-glutamine, and 1% P/S on 75-cm^2^ adherent cell culture flasks (Gibco, Germany). The medium was changed with fresh medium every 3–4 days. When the cells reached ~ 80% confluence, the cells were then detached using 0.05% Trypsin/EDTA (Gibco, Germany) and re-cultured into 75-cm^2^ flasks at a density of 6 × 10^5^ cells/flask.

### Characterization of MSCs

To analyze the immune profile of MSCs, flow cytometry was used as described by the International Society for Cellular Therapy (Yang et al. 2017). MSCs were stained with directly fluorescein isothiocyanate (FITC) conjugated antibodies against CD105, CD34, and CD45 (Abcam, USA). Stained cells were analyzed on FACS flow cytometry using Cell Quest Software (Becton Dickinson, UK).

### Preparation of scaffold

R-GSIK was custom synthesized by CPC Scientific (Purity > 98%, GL Biochem, China). High-performance liquid chromatography was performed to identify the purity of the peptides. The aqueous peptide solution was prepared using Milli-Q water (18.2 MX), stored at 4 °C, and sonicated for 30 min before application.

### TBI model and experimental groups

Forty-five male Wistar rats (200 ± 20 g) were purchased from Animal Center of Mashhad University of Medical Sciences. Animals were kept under a 12:12 day/night cycle and given ad libitum access to food and water. The animals were anesthetized with an intraperitoneal injection of ketamine (80 mg/kg) and xylazine (20 mg/kg). Next, each animal’s scalp was shaved and disinfected using 70% ethanol. Skulls were fixed in a stereotactic frame. After incising the skin and retracting fascia with sterilized gauze, a square-shaped part of the skull was removed (AP = 2 mm; ML = − 1 mm) using a dental micro drill. A punch biopsy (2-mm diameter) was attached to the drill and removed a small portion of the brain (AP = 0 mm; ML = − 1.5 mm; DV = − 2 mm: M1/M2 area). Animals were divided into five groups (Table 1). No treatment was performed in the TBI group (control group). PBS, R-GSIK, MSCs, and MSCs+R-GSIK groups were treated with PBS (vehicle), R-GSIK (nano-scaffold), MSCs, and MSCs+R-GSIK, respectively. The live cells (5 × 10^5^) were diluted in PBS (10 μl) and then transplanted by a Hamilton syringe. During the experiments, animals were monitored for mortality/viability (4 of 45 rats died after the injury).

**Table 1:** Summary of different experimental groups

Groups	Intervention	Number of animals
TBI	TBI without any treatment	8
PBS	TBI-received PBS (vehicle)	8
R-GSIK	TBI-received R-GSIK	8
MSCS	TBI-received MSCS	8
MSCs+R-GSIK	TBI-received MSCs+R-GSIK	9

### Behavioral assessment

To assess whether treatment with MSCs and R-GSIK can promote functional improvements, we performed mNSS, OF, and EPM on various days after the injury. To assess the motor and sensory functions, mNSS test was performed on 1, 7, 14, 21, and 28 days after TBI, as described previously (Sahab Negah et al. 2019).

To analyze general motor activity, OF was conducted on day 29 after TBI. The total distance was recorded by a video camera for 5 min. Each trial was recorded and analyzed using Borj-Sannat Tracking software (Tehran, Iran). The arena was cleaned by 70% ethanol between each trial to remove odor.

The EPM test was performed to measure anxiety-like behavior on day 30 post-injury. The EPM apparatus consisted of two open arms and two plastic black closed arms. Each animal was placed at the center facing the open arm. Each rat activity was recorded by a video camera for 5 min, and time in open arm and open arm entries were recorded and analyzed via Borj-Sanaat Tracking software (Tehran, Iran). The platforms were cleaned before testing by 70% ethanol.

### Immunohistochemistry

Immunohistochemistry on paraffin-embedded sections was conducted, as described previously (Sahab-Negah et al. 2020). Briefly, brain tissues were fixed in 4% paraformaldehyde and embedded in paraffin. The thickness of the tissues was cut into 5-μm sections. After deparaffinization, brain sections were boiled in 10 mM citric acid buffer (pH = 6) for 10 min. Sections were incubated with normal goat serum (Sigma, Germany) containing 0.3% Triton-X-100 in PBS. Sections were then incubated with primary antibodies, including mouse anti-glial fibrillary acidic protein (GFAP), a marker for reactive astrocytes (1:500; Abcam, UK); rabbit anti-ionized calcium-binding adapter molecule 1 (Iba1) antibody (1:1000; Wako) to encounter microglia at 4 °C overnight. Horseradish-peroxidase (HRP)–conjugated anti-mouse antibody (1:100; Abcam) and anti-rabbit antibody (1:500, Abcam) was added to sections at room temperature for 1 h. For negative controls, primary antibodies were deleted. The sections were visualized by 3,3′-diaminobenzidine and analyzed with a bright field microscope.

### Western blotting

Tissues were washed by PBS and mashed in a mortar. The cell suspension was transferred and centrifuged (for 15 min at 4 °C) after adding cell lysis buffer (0.1 M NaCl, 0.01 M Tris, 0.1 mM EDTA) and a protease inhibitor. The supernatant was used to determine total protein concentration using the standard Bradford method. The protein was transferred to a polyvinylidene fluoride membrane for 1 h. Proteins were detected by incubation with primary antibodies against toll-like receptor 4 (TLR-4), tumor necrosis factor-alpha (TNF), and interleukin 6 (IL-6) at a dilution of 1∶500 (Santa Cruz, Germany) followed by secondary antibodies HRP-conjugated (1:1000, Santa Cruz, Germany). Finally, Western blot analyses were detected using the chemiluminescence kit (Fermentase, Germany). Results were quantified using ImageJ software.

### Statistical analyses

The analysis of variance (ANOVA) was used for repeated measurements of the mNSS test. For other dependent variables, one-way ANOVA followed by post hoc Tukey’s tests was used to compare the differences between groups. Data are represented as the means ± standard deviations (SD). *P* < 0.05 was regarded as statistically significant.

## Results

### Characterization of stem cells

**Fig. 1:**
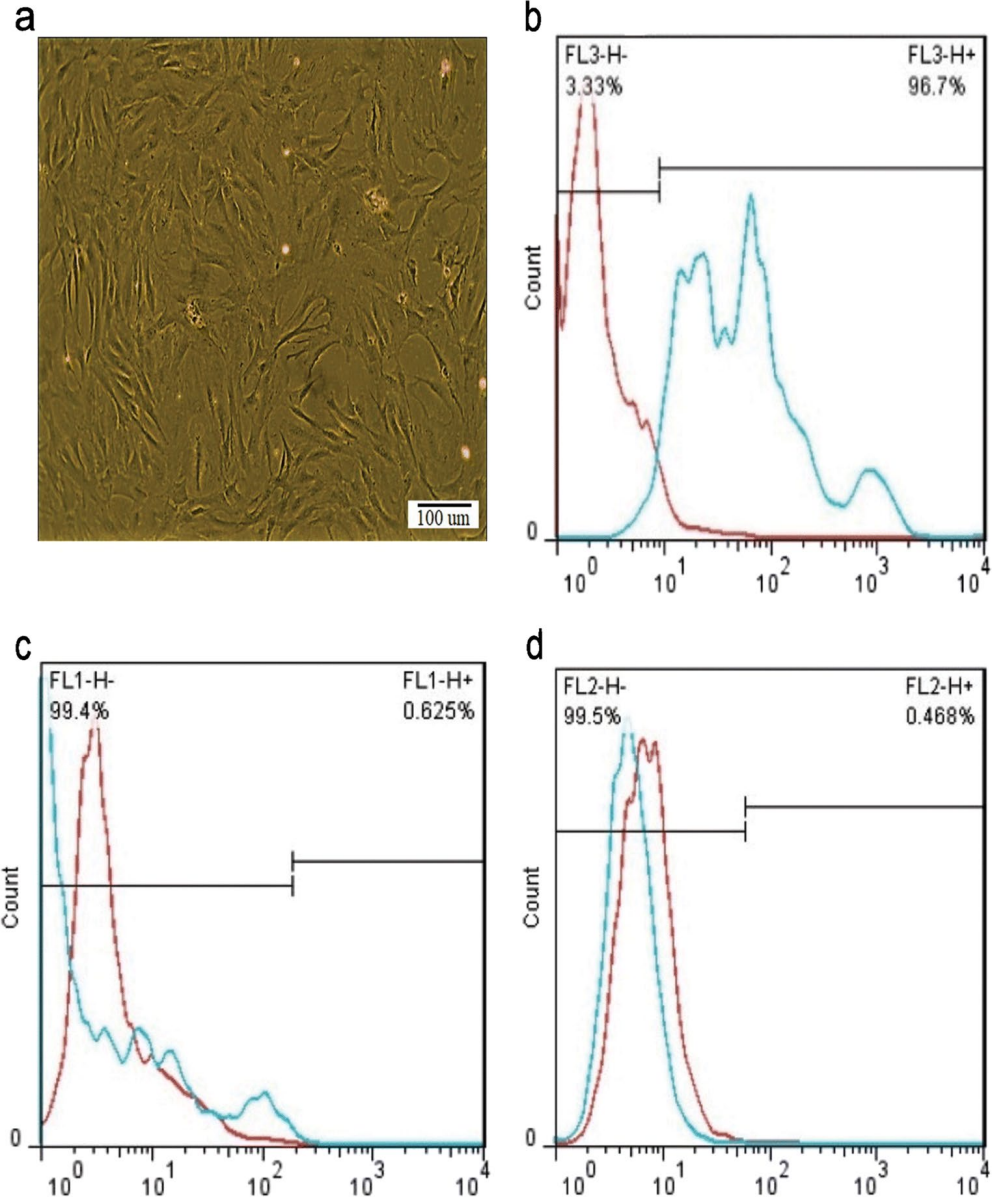
MSCs characterization by flow cytometry. **a**) Phase-contrast micrographs of MSCs derived from adipose tissue in passage 4. **b**-**d**) The cells were positive for CD105 as a MSCs marker (**b**), and negative for the CD45 (**c**) and CD34 (**d**) as the hematopoietic markers

To characterize the MSCs, specific markers CD105, CD34, and CD45 were analyzed using flow cytometry. The results showed that MSCs expressed CD105, a mesenchymal marker, but did not express CD34 and CD45, the hematopoietic markers (Fig. 1).

### Sensory-motor function

**Fig. 2:**
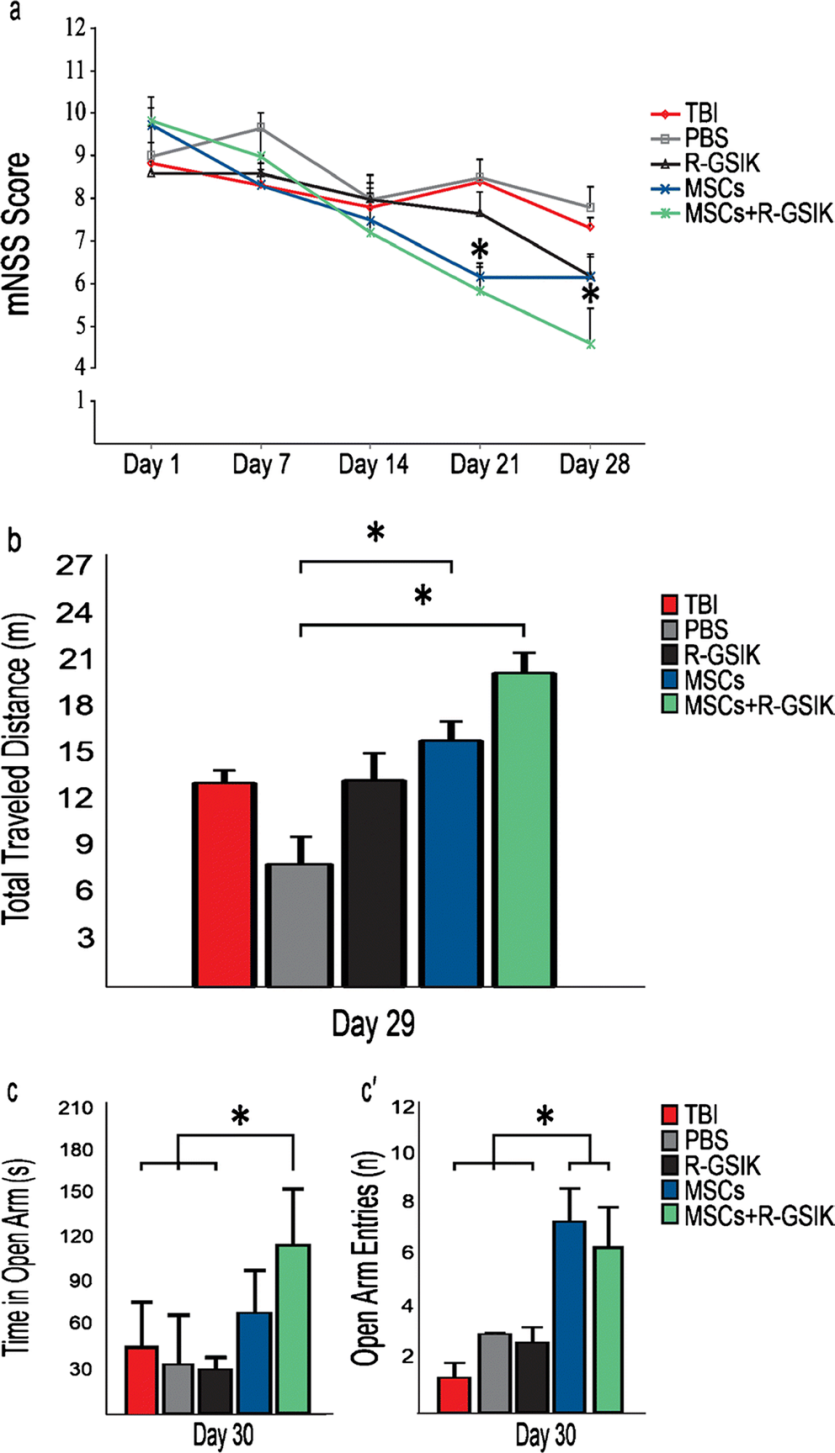
Sensory-motor function, general activity, and anxiety-like behavior were performed in different groups. (**a**) Modified neurological severity scores (mNSS) were performed in different groups. mNSS significantly improved in rats treated with MSCs+R-GSIK and MSCs compared with TBI and PBS groups on day 21 after TBI. We also observed that mNSS significantly decreased on day 28 in MSCs+R-GSIK group compared with control groups. *P < 0.05 vs. TBI and PBS. (**b**) Evaluation of traveled distance in the open field on day 29 post-TBI. In the open-field task, traveled distance significantly increased in MSCs+R-GSIK and MSCs groups in comparison with the PBS group. (**c**-**c**') Evaluation of time in open arm and open arm entries in the elevated plus-maze on day 30 after TBI. (**c**) Time in open arm significantly increased in MSCs+R-GSIK group compared with control groups. (**c**') We also observed that open arm entries significantly increased in MSCs+R-GSIK and MSCs groups compared with control groups. Data are presented as mean ± SD. *P < 0.05

Our results showed that mNSS was significantly reduced in the MSCs+R-GSIK group on days 21 and 28 after the treatment compared with the TBI and PBS groups (Fig. 2a; *P* < 0.05). The neurological deficit test also indicated that MSCs treatment significantly improved the mNSS compared with the TBI and PBS groups on day 21 (Fig. 2a; *P* < 0.05).

### General activity and anxiety like behavior

To determine the general activity level and to measure anxiety-like behavior, the OF and EPM were performed, respectively. In the OF task, a statistically significant difference in total traveled distance was revealed between the MSCs+R-GSIK and MSCs groups with the PBS group (Fig. 2b; *P* < 0.05). The time spent in the open arms in the EPM test significantly increased in the MSCs+R-GSIK group compared with the TBI, PBS, and R-GSIK groups (Fig. 2c; *P* < 0.05). Open arm entries in the EPM task also significantly increased in the MSCs+R-GSIK and MSCs rats compared with the other groups (Fig. 2c'; *P* < 0.05).

### Immunohistochemistry

**Fig. 3:**
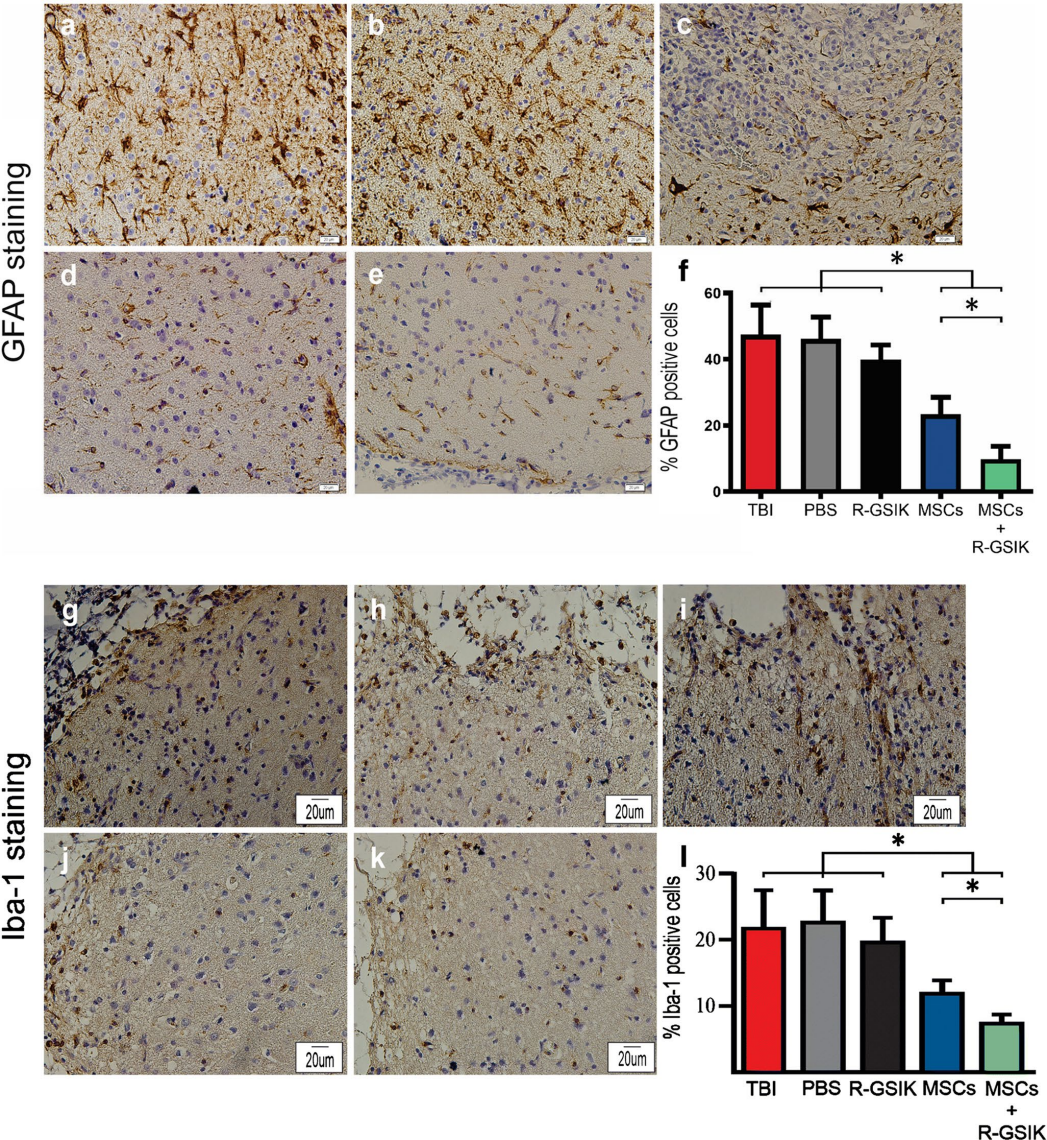
Representative immunohistochemistry (IHC) images show the expression of GFAP and Iba-1 (brown cells) within the injury site. Bar graphs show the mean number of GFAP- and Iba1-positive cells in the lesion site 30 days after TBI in different animal groups. Administration of MSCs+R-GSIK and MSCs decreased the number of GFAP- and Iba-1-positive cells within the injury site compared with the control groups. Data are expressed as mean ± SD. *P < 0.05

We used immunohistochemistry to identify Iba-1 and GFAP markers to assess the therapeutic effect of MSCs treatment on the quantity of astrogliosis and reactive microglia. Transplantation of MSCs seeded in R-GSIK significantly decreased the mean percentage of GFAP-positive cells around the injury site compared with the MSCs group (Fig. 3a-f; *P* < 0.05). Furthermore, a significantly lower number of Iba-1-positive cells were detected in the MSCs+R-GSIK group compared with the MSCs-only group (Fig. 3g-l; *P* < 0.05). In addition, the mean percentages of GFAP- and Iba-1-positive cells in the MSCs+R-GSIK and MSCs groups were significantly lower than those in the TBI, PBS, and R-GSIK groups (Fig. 3f and l; *P* < 0.05).

### Inflammatory cytokines

**Fig. 4:**
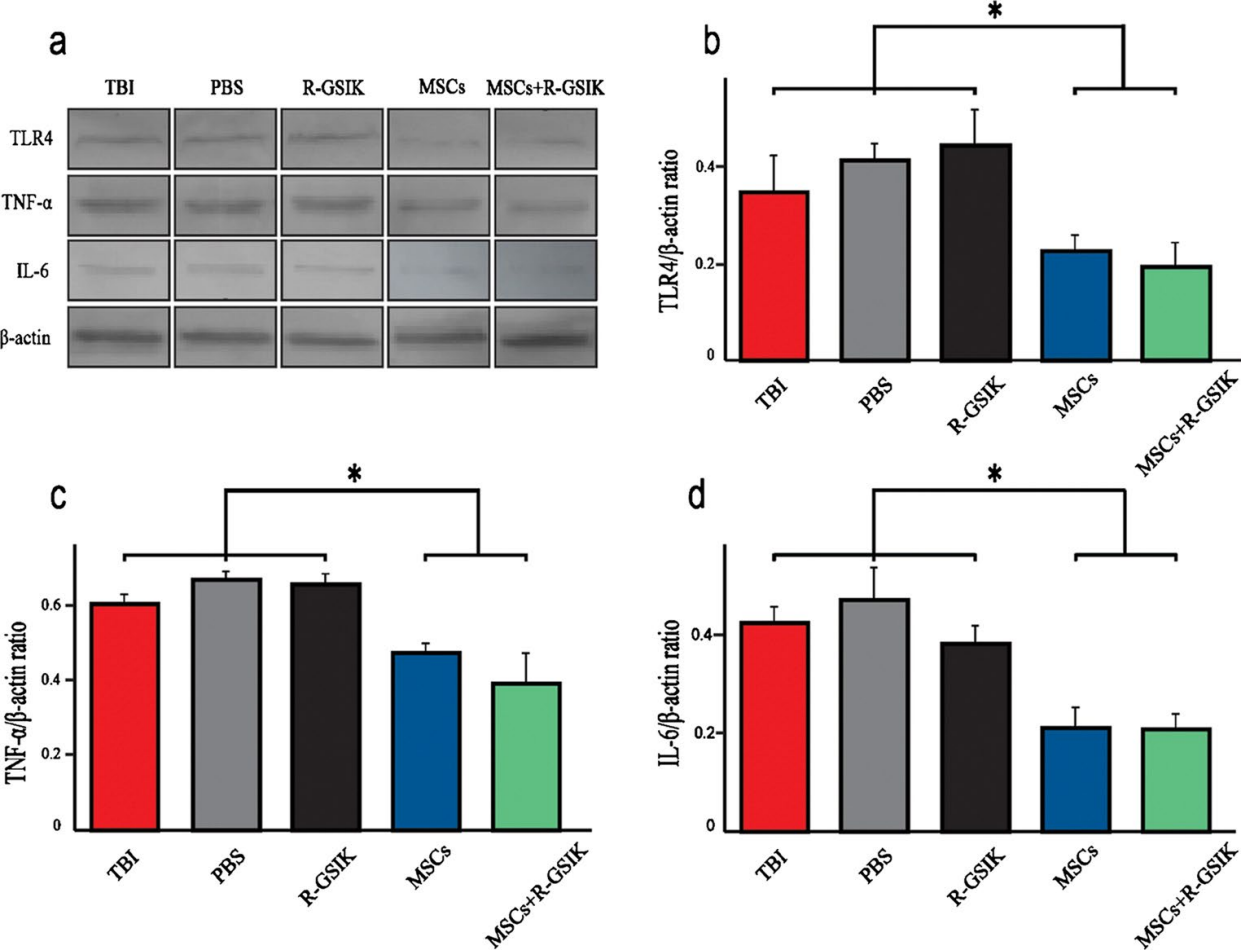
Evaluation of the protein levels of TLR4, TNF, and IL6 was analyzed on day 30 after induction of traumatic brain injury (TBI). Our results showed that the protein levels of TLR4, TNF, and IL6 significantly decreased in the MSCs+R-GSIK and MSCs groups compared with the TBI, PBS, and R-GSIK groups. Densitometry of the markers bands correlated to the β-actin band. The data are shown as mean ± SD. *P < 0.05

To elucidate potential anti-inflammatory effects of MSCs combined with R-GSIK, we analyzed pro-inflammatory cytokines, including TLR-4, IL6, and TNF (Fig. 4a). The protein level of TLR4 was significantly lower in the MSCs and MSCs+R-GSIK groups than the control groups (Fig. 4b; *P* < 0.05). The level of TNF was also significantly lower in the MSCs and MSCs+R-GSIK rats than the other groups (Fig. 4c; *P* < 0.05). Besides, the protein level of IL-6 was significantly lower in the MSCs and MSCs+R-GSIK groups than the other groups (Fig. 4d; *P* < 0.05).

## Discussion

 Our findings indicate that co-transplantation of R-GSIK with MSCs significantly improved neurological deficits and anxiety-like behaviors. We also demonstrated that MSCs seeded in R-GSIK significantly decreased pro-inflammatory cytokines and gliosis within the injury site.

Stem cell therapy has emerged as an effective treatment option for diverse neurological diseases (Wang et al. 2018). Patients suffering from TBI may benefit from this novel therapeutic strategy (Hasan et al. 2017; Weston and Sun 2018). The inflammatory reactions at the injury site after TBI lead to a lower survival rate and reduce the success of cell transplantation (Cox 2018). There is growing interest in the application of MSCs in regenerative medicine as they can obtain from various tissues and differentiate into neural cells (Sanchez-Ramos et al. 2000). Furthermore, MSCs migrate to the injured site and help the regeneration through the reduction of immune response and release of growth factors (Camberlain et al. 2007; Galindo et al. 2011; Parr et al. 2007). The clinical application of MSCs is restricted as these cells need a well-established and reliable grafting technique (Hasan et al. 2017). Biomaterial scaffolds have been introduced as a promising method to overcome this issue (Shi et al. 2016; Sahab Negah et al. 2018). Scaffolds should be accompanied by appropriate biocompatibility, mechanical properties, and biodegradability in vivo after implantation (Zhou et al. 2018). Here, we have shown that the beneficial effects of MSCs were improved when engrafted with R-GSIK as a nano-scaffold in a TBI model.

The mNSS scoring method is used to evaluate animal recovery after brain injury and is a composite of motor, sensory, reflex, and balance tests (Guo et al. 2013). This study found that the MSC-loaded R-GSIK scaffold enhanced the TBI recovery, as the MSCs+R-GSIK group showed a significantly lower mNSS than the control groups (Sahab Negah et al. 2019; Zhang et al. 2018; Peng et al. 2015). In addition, our observations showed that the combination treatment of MSCs+R-GSIK significantly recovered the anxiety-like behavior. This study supports evidence from a previous observation (Darkazalli et al. 2016). These results support our previous research into this brain area which R-GSIK improved the function of human meningioma stem-like cells (Sahab Negah et al. 2019).

Neuroinflammation plays an important role in the pathological process  of the secondary injury responses after TBI (Kumar and Loane 2012). Inflammatory-related molecules, such as pro- and anti-inflammatory cytokines, are primarily released by activating astrocytes and microglia after TBI (Karve et al. 2016). Furthermore, activated astrocytes proliferate and cause impairment in axonal regrowth (Laird et al. 2008). On the other hand, the ablation of reactive astrocytes increases leukocyte infiltration at the injury site and leads to neurodegeneration (Bush et al. 1999). Based on the dual role of astrocytes, regulation of inflammatory responses of astrocytes therapeutically conducts TBI immunity toward a promising response that might repair brain functions (Jassam et al. 2017). Zhang and colleagues reported that the densities of astrocytes were not significantly different after the application of MSCs in a TBI rat model (Zhang et al. 2013). In contrast to earlier findings, however, MSCs significantly decreased astrocytes within the injury site. Interestingly, we also observed that MSCs+R-GSIK significantly decreased the number of astrocytes compared with the MSCs group.

Another significant aspect of neuroinflammation is microglia that mainly produce pro-inflammatory cytokines, such as TNF and IL-6, which activate glial cells and induce further cytokine production and astrogliosis (Ziebell and Morganti-Kossmann 2010; Konsman et al. 2007). Pro-inflammatory cytokines are also regulated by TLR4 as a key host molecule in the regulation of the innate immune response (Ahmad et al. 2013; Ashayeri Ahmadabad et al. 2020). Thus, inhibition of activated microglia and TLR4 can decrease inflammation and increase histological and functional outcomes after TBI (d’Avila et al. 2012; Feng et al. 2017). In this study, we have shown that co-engraftment of MSCs with R-GSIK after TBI is associated with a lower number of microglia, decreased expression of TLR4, and reduced levels of pro-inflammatory cytokines TNF and IL-6. This study confirms that the combination therapy of stem cells with a self-assembling peptide improves the inflammatory response (Sahab Negah et al. 2019; Shi et al. 2016; Wang et al. 2015).

To sum up, our findings indicated that MSCs and R-GSIK improve sensory-motor recovery and attenuate inflammatory response after TBI. Additional investigations are needed to guide further clinical studies.
