# Correction to: Transplantation of R-GSIK scaffold with mesenchymal stem cells improves neuroinflammation in a traumatic brain injury model

**DOI:** 10.1007/s00441-025-03991-1

**Published:** 2025-06-30

**Authors:** Sajad Sahab Negah, Mohammad Moein Shirzad, Ghazale Biglari, Farzin Naseri, Hassan Hosseini Ravandi, Ali Hassani Dooghabadi, Ali Gorji

**Affiliations:** 1 Neuroscience Research Center, Mashhad University of Medical Sciences, Mashhad, Iran; 2 Department of Neuroscience, Faculty of Medicine, Mashhad University of Medical Sciences, Mashhad, Iran; 3 Shefa Neuroscience Research Center, Khatam Alanbia Hospital, Tehran, Iran; 4 Student Research Committee, Mashhad University of Medical Sciences, Mashhad, Iran; 5 Department of Neurosurgery and Department of Neurology, Westfälische Wilhelms-Universität, Münster, Germany; 6 Epilepsy Research Center, Westfälische Wilhelms-Universität Münster, Münster, Germany

**Correction to: Cell and Tissue Research (2020) 382:575–583**

10.1007/s00441-020-03247-0

The authors regret that the version of Figure 3 that appeared in the original published article is incorrect.

An error occurred during the assembly of the upper portion of Figure 3, specifically in the panels displaying GFAP staining. Images originating from the staining setup process were inadvertently included in the panels representing experimental groups. As a result, two sets of images in the GFAP panels partially overlapped, leading to incorrect representation of different experimental groups. Figure 3 has now been corrected to display the appropriate representative images corresponding to each experimental group in the GFAP staining section. All associated quantitative analyses have been carefully reviewed and confirmed to be based on the correct underlying data. The main results and conclusions remain unaffected. Both the original and corrected versions of Figure 3 are provided below. We sincerely apologize for this oversight and any confusion it may have caused.
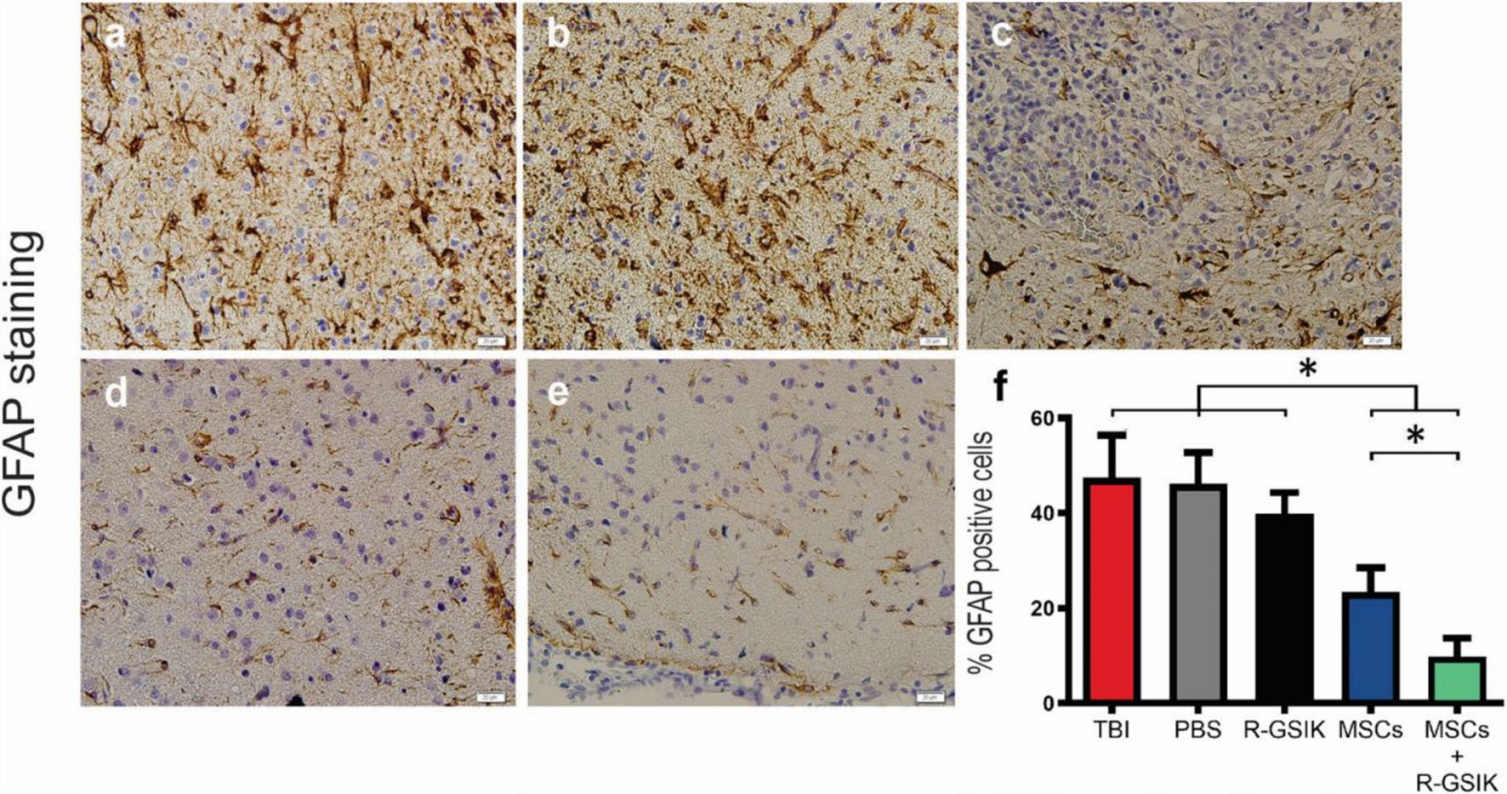


**Correct Figure 3 (upper panel).** Representative immunohistochemistry (IHC) images show the expression of GFAP (brown cells) within the injury site. Bar graphs show the mean percentage of GFAP-positive cells in the lesion site 30 days after TBI in different animal groups. Administration of MSCs+R-GSIK and MSCs decreased the number of GFAP- positive cells within the injury site compared with the control groups. Data are expressed as mean ± SD. *P < 0.05
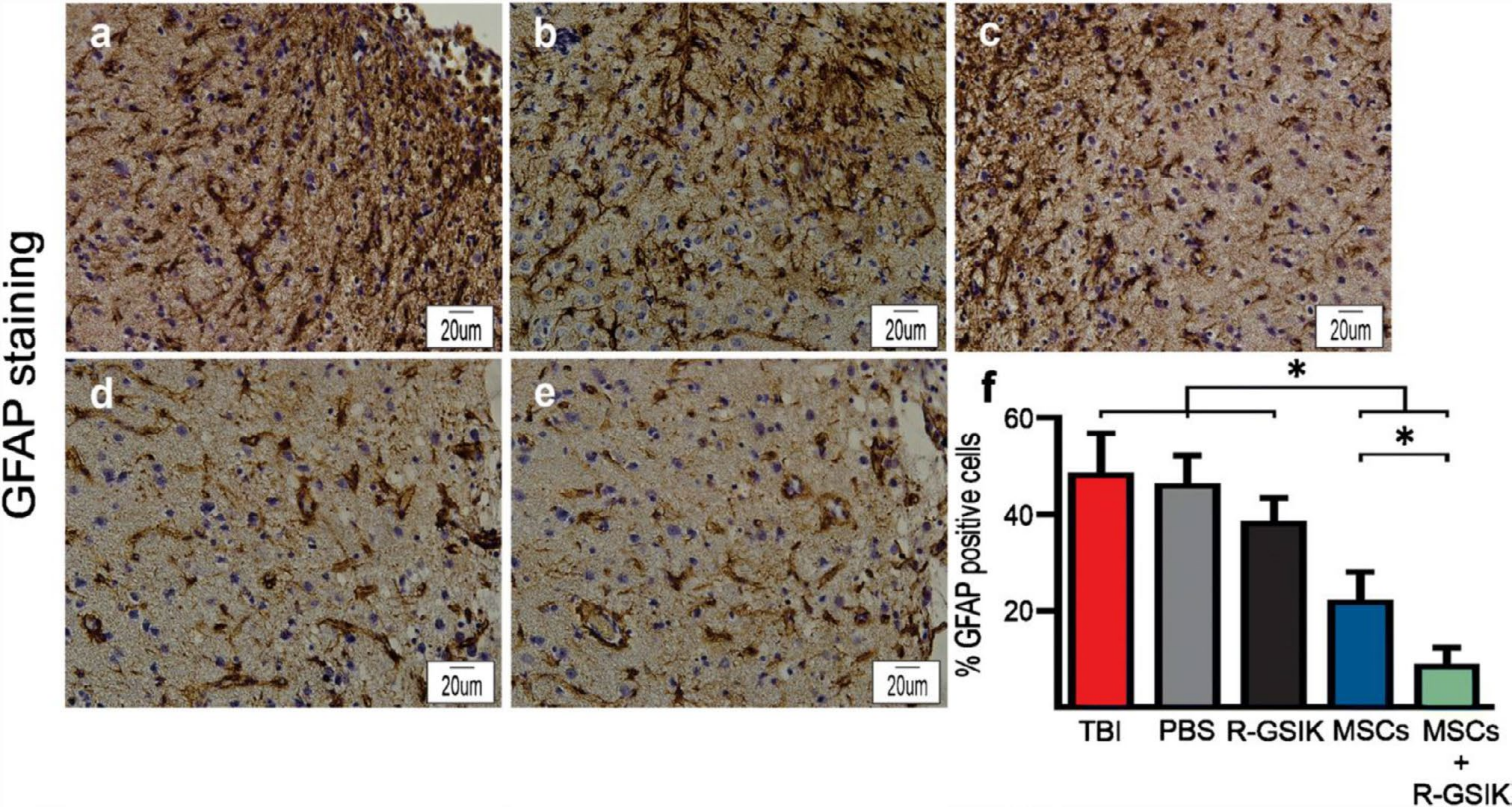


**Incorrect Figure 3 (upper panel).** Representative immunohistochemistry (IHC) images show the expression of GFAP (brown cells) within the injury site. Bar graphs show the mean number of GFAP-positive cells in the lesion site 30 days after TBI in different animal groups. Administration of MSCs+R-GSIK and MSCs decreased the number of GFAP-positive cells within the injury site compared with the control groups. Data are expressed as mean ± SD. *P < 0.05

The original article has been corrected.

The original article can be found at https://doi.org/10.1007/s00441-020-03247-0.

